# Cross-sectional Evaluation of the Mechanical Axis and Other Angular Parameters of the Lower Limbs in Male Professional Soccer Players

**DOI:** 10.1055/s-0046-1818613

**Published:** 2026-04-22

**Authors:** Jean Klay Santos Machado, Eurineto Gomes do Nascimento, Erik Silva de Menezes

**Affiliations:** 1 Orthopedics and Traumatology Service, Hospital Porto Dias, Belém, PA, Brazil

**Keywords:** biomechanical phenomena, soccer, sports medicine, fenômenos biomecânicos, futebol, medicina esportiva

## Abstract

**Objective:**

To evaluate the mechanical axis and angular parameters of the lower limbs in professional soccer players, relating them to age, limb dominance, and field position.

**Methods:**

The present cross-sectional study included 102 male athletes from a Brazilian professional soccer team. Full-length radiographs in the orthostatic position were analyzed using the PeekMed (Peek Health, S.A.) software to measure the condylar efficiency (CE), the medial mechanical proximal tibial angle (mMPTA), the mechanical lateral distal femoral angle (mLDFA), and the mechanical tibiofemoral angle (mTFA). The results were analyzed using descriptive statistics, analysis of variance (ANOVA), multivariate analysis of variance (MANOVA), Pearson's/Spearman's correlations, and multiple regression.

**Results:**

The mean age of the participants was of 26.9 years. The valgus pattern was the most prevalent (68.6%), with bilateral valgus in 47.0% of the sample. A negative correlation was found between age and bilateral CE (ρ = -0.42 on the right side; and -0.39 on the left side; *p* < 0.05), and defenders and left-footed players presented greater condylar asymmetry (ΔCE). The MANOVA indicated a significant association between field position and angular parameters ( *p* = 0.0016), with no global effect of dominance ( *p* = 0.243). Midfielders and fullbacks showed a higher prevalence of valgus (of up to 80%), while defenders and goalkeepers commonly presented with neutral alignment. There were significant bilateral differences in CE, mMPTA, and mTFA.

**Conclusion:**

Age and field position influenced lower-limb alignment in professional soccer players, with more pronounced asymmetries in defenders and left-footed athletes. Individualized monitoring of the mechanical axis may help prevent injuries and optimize performance.

## Resumo

**Objetivo:**

Avaliar o eixo mecânico e os parâmetros angulares dos membros inferiores em atletas profissionais de futebol, relacionando-os à idade, dominância lateral e posição em campo.

**Métodos:**

Conduzimos um estudo transversal com 102 atletas masculinos de um clube. Radiografias panorâmicas em ortostase foram analisadas pelo programa PeekMed (Peek Health, S.A.), que mensurou o parâmetro de eficiência condilar (PEC), o ângulo mecânico medial proximal tibial (AmMPT), o ângulo mecânico lateral distal femoral (AmLDF) e o ângulo mecânico tibiofemoral (AmTF). Aplicaram-se estatística descritiva, analise de variância ( *analysis of variance* , ANOVA, em inglês), análise multivariada da variância ( *multivariate analysis of variance* , MANOVA, em inglês), correlações de Pearson/Spearman e regressão múltipla.

**Resultados:**

A idade média foi de 26,9 anos. O valgo foi o padrão mais prevalente (68,6%), com bilateralidade em 47,0%. Houve correlação negativa entre idade e PEC bilateral (ρ = -0,42 à direita; e -0,39 à esquerda) e maior assimetria condilar (ΔPEC) em zagueiros e canhotos. A MANOVA indicou associação significativa entre posição e parâmetros articulares ( *p* = 0,0016), sem efeito global da dominância ( *p* = 0,243). Laterais e meias apresentaram maior prevalência de valgo (até 80%), ao passo que zagueiros e goleiros mostraram mais casos de eixo neutro. Diferenças bilaterais significativas foram observadas com relação ao PEC, ao AmMPT e ao AmLDF.

**Conclusão:**

O alinhamento dos membros inferiores em atletas de futebol mostrou-se influenciado pela idade e posição em campo, com assimetrias mais marcantes em zagueiros e canhotos. O monitoramento individualizado do eixo mecânico pode auxiliar na prevenção de lesões e otimização do desempenho.

## Introduction

Coronal alignment of the lower limbs is fundamental in sports orthopedics, particularly for athletes exposed to high biomechanical loads. Mechanical-axis alterations can result in joint overload, increase susceptibility to injury, and compromise performance. Although the traditional knee classification includes neutral, varus, or valgus categories, it does not reflect the individual anatomical complexity. 1

A neutral knee corresponds to a hip-knee-ankle (HKA) angle of 180°, with oblique femoral and tibial joint lines at 3° (tibial varus and femoral valgus), resulting in a femoral mechanical angle (FMA) of 93° and a tibial mechanical angle (TMA) of 87°, both measured medially. 1

Anatomical and mechanical factors, primarily the femoral and tibial joint lines, determine alignment. 2 Key alignment parameters include the lateral anatomical distal femoral angle (LaDFA), the medial proximal tibial angle (MPTA), and the quadriceps (Q) angle, which are relevant indicators in the evaluation of straight alignment in soccer players. 3 Variations in these angles can predispose to injuries and compromise athletic performance. 3

Mechanical axis deviation (MAD), assessed by panoramic radiography, can generate asymmetrical overload in the knee. Varus (medial axis) is associated with lesions to the medial compartment, while valgus (lateral axis) is related to lateral lesions. 4 5 Panoramic radiography is the gold standard, providing objective and reproducible measurements. 6 Accuracy depends on the correct identification of anatomical landmarks. 7 The mechanical axis is a central concept in knee biomechanics, influencing the distribution of joint loads and the risk of injury. Its recognition and correction are fundamental in the prevention and treatment of sports injuries. 4 6

Based on these considerations, the current study aimed to evaluate the mechanical axis and other angular parameters of the lower limbs in male professional soccer players, relating them to age, lateral dominance, and position on the field.

## Methods

### Ethical Aspects

The institutional Ethics in Research Committee approved the present study in accordance with the Declaration of Helsinki, the Nuremberg Code, and Resolution no. 466/12 of the Brazilian National Health Council (Conselho Nacional de Saúde, CNS, in Portuguese) under number CAAE 84206924.5.0000.0235. All participants signed the informed consent form. Potential risks, such as of data leakage and exposure in a hospital setting, were minimized through anonymization of information, secure storage of records, and strict adherence to biosafety standards recommended by the World Health Organization. As a benefit, the study expanded knowledge about mechanical alignment in professional athletes, supporting future research and preventive interventions.

### Participants, Study Site, and Sample Size Rationale

The study was conducted in a high-complexity hospital in the Northern Region of Brazil, between January 2023 and December 2024, with 102 professional male soccer players, including all the club's players during that period. The sample represented the target population of high-performance athletes, and it was suitable for multivariate analyses (analysis of variance [ANOVA], multivariate ANOVA [MANOVA], multiple regression, and correlations), consistent with previous studies including 30 to 100 subjects.

The sample size ( *n* = 102) was evaluated for statistical power using the R software (R Foundation for Statistical Computing), with the pwr package, in a multiple regression model with 3 predictors (age, position, and lateral dominance). The analysis considered a significance level of 5% (α = 0.05) and a moderate effect size (F ^2^ = 0.15), according to Cohen's criteria. 8 The results indicated that the sample had adequate statistical power to detect associations of moderate magnitude regarding the variables analyzed, meeting the methodological robustness criteria recommended in the literature.

### Data Collection and Analysis

Data collection took place between January 2023 and December 2024 via an electronic questionnaire (Google Forms, Alphabet Inc.) and standardized radiographic examinations, totaling 102 athletes with complete responses. Participants with incomplete forms or flaws in the radiographic stages were excluded from the study, with no missing data in the final sample. The information was organized in spreadsheets using Microsoft Excel 2016 (Microsoft Corp.).

The analysis of the mechanical axis of the lower limbs used panoramic radiographs in orthostasis with bipodal support. These radiographs were processed by an orthopedist member of the Brazilian Society of Orthopedics and Traumatology (Sociedade Brasileira de Ortopedia e Traumatologia, SBOT, in Portuguese) and the Brazilian Society of Knee Surgery (Sociedade Brasileira de Cirurgia de Joelho, SBCJ, in Portuguese), with the aid of the PeekMed (Peek Health, S.A.) software. The process involved calibration with a 25-mm metallic sphere and identification of anatomical landmarks (femoral head, tibial plateau, and talus). The mechanical axis was traced from these points, enabling the objective analysis of varus or valgus misalignments. A range from 0° to 2° was considered normal for both valgus and varus.

**Fig. 1:**
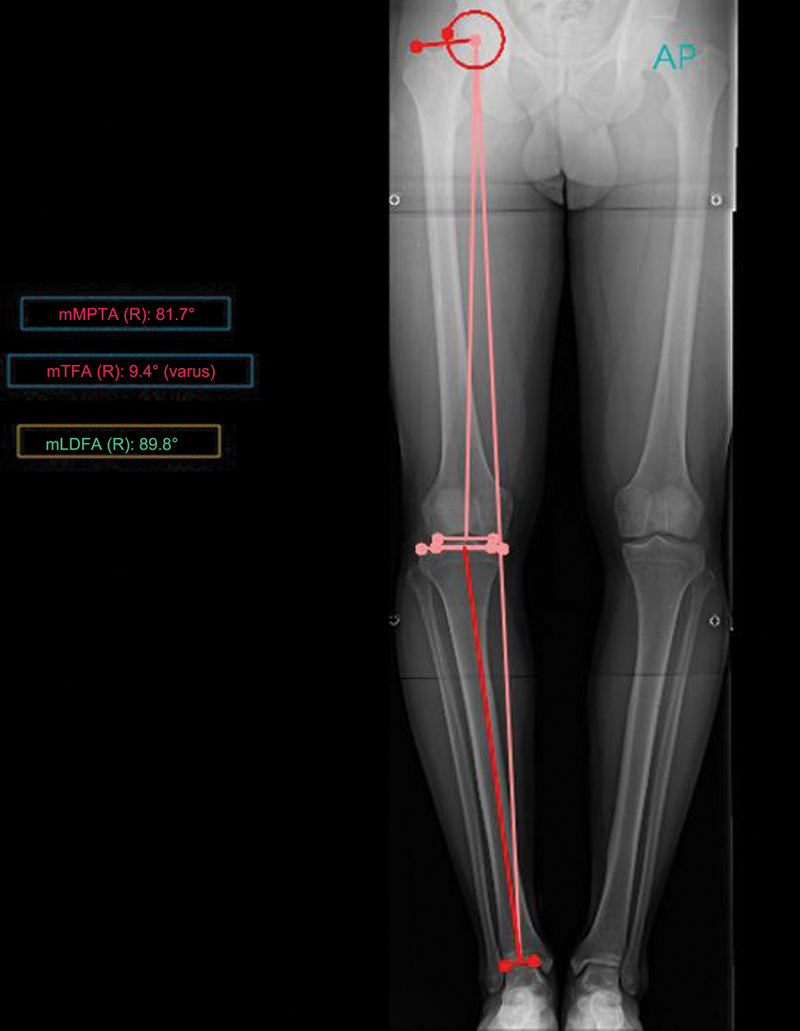
Lower limb panoramic radiograph with angular measurements. **Abbreviations:** AP, anteroposterior; mLDFA, mechanical lateral distal femoral angle; mMPTA, medial mechanical proximal tibial angle; mTFA, mechanical tibiofemoral angle; R, right side.

PeekMed automatically provided measurements for each lower limb, as described in Figs. 1 2 :

**Fig. 2:**
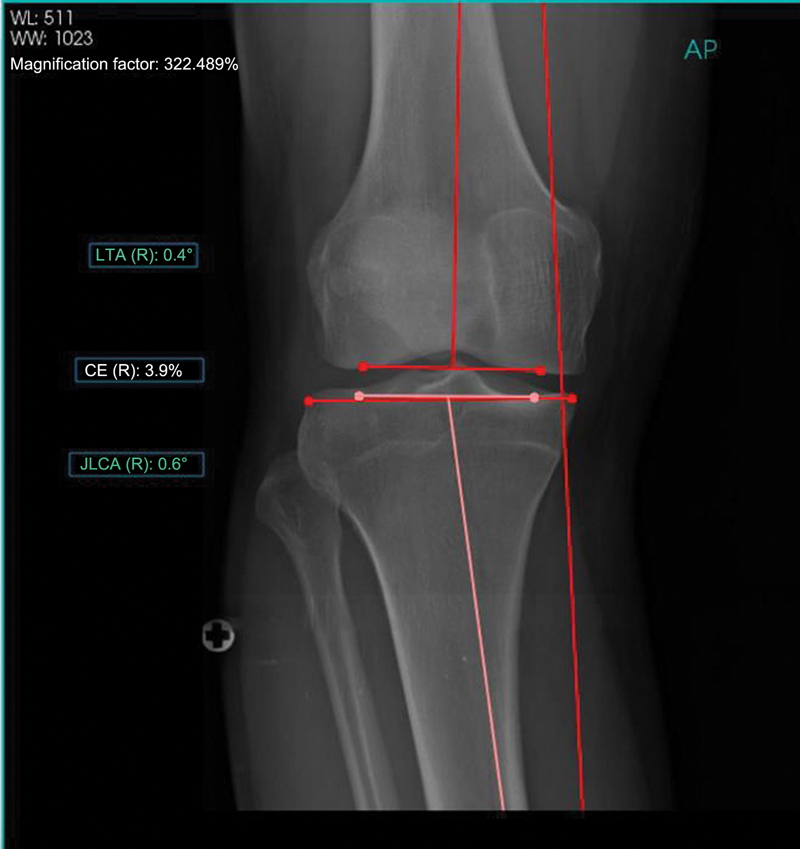
Anteroposterior knee radiograph with angular measurements. **Abbreviations:** AP, anteroposterior; CE, condylar efficiency; JLCA, joint line congruence angle; LTA, lateral tilt angle; R, right side.

- MAD in millimeters;
- HKA (between the femoral and tibial mechanical axes);
- Lateral distal femoral angle (LDFA); and
- Medial proximal tibial angle (MPTA).

All measurements were digitally recorded, exported, and analyzed using the PeekMed software, ensuring measurement accuracy and reproducibility. The variables were expressed as mean, median, and standard deviation values. The normality of the sample was evaluated using the Shapiro-Wilk test. Pearson's or Spearman's correlation coefficients were applied as appropriate. Group comparisons were conducted using MANOVA, ANOVA, or the Kruskal-Wallis test based on data distribution. Multiple regression models examined the influence of age, field position, and dominance on angular outcomes. Paired analyses comparing the right and left sides were performed using the Student's t-test or the Wilcoxon signed-rank test. The statistical significance level was of 5% ( *p* < 0.05).

## Results

The current study evaluated 102 professional soccer players, with a mean age of 26.9 ± 5 years, a typical age range for athletes in full activity. Of these, 81% kicked with their right foot (right-footed) and 19%, with their left foot (left-footed). Regarding their field position, 31% were forwards, 21%, midfielders, 18%, defenders, 15%, full-backs, 12%, defensive midfielders, and 4%, goalkeepers.

**Fig. 3:**
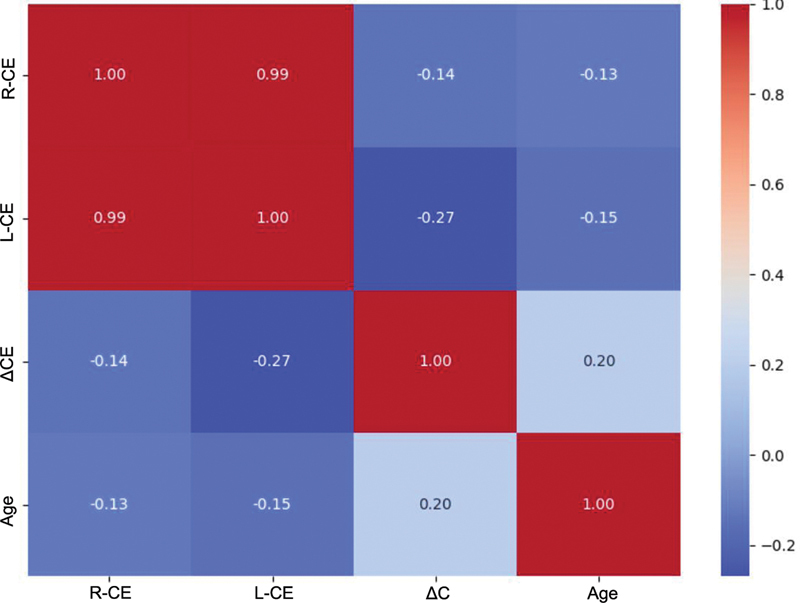
**Correlogram showing Spearman's correlation coefficients between age and condylar variables (right-sided condylar efficiency [R-CE], left-sided condylar efficiency [L-CE], and condylar asymmetry [ΔCE]).** Coefficients are displayed within the cells, with color intensity proportional to the strength of the correlation.

The Spearman's correlation demonstrated a negative association between age and condylar efficiency (CE) in both the right (ρ = − 0.42; *p* = 0.002) and left knees (ρ = − 0.39; *p* = 0.005), indicating lower CE values in older athletes ( Fig. 3 ). The CE reflects the alignment and efficiency of the femur-tibia contact, with reduced values suggesting a higher risk of joint imbalance. The left tibiofemoral angle (LTFA) progressively increased with age (ρ = +0.28; *p* = 0.01), suggesting potential knee alignment adaptation or overload over time ( Table 1 ).

**Table 1:** Correlation between age and angles

Variable	Coefficient (r/ρ)	*p* -value	Type
R-CE	-0.42	0.002	Spearman
L-CE	-0.39	0.005	Spearman
L-mTFA	+0.28	0.01	Spearman

Abbreviations: L-CE, left-sided condylar efficiency; L-mTFA, left-sided mechanical tibiofemoral angle; R-CE, Right-sided condylar efficiency.

Multiple linear regression for right-sided CE identified the following 3 significant predictors: age (β = -0.38; *p* = 0.004), left-handed dominance (β = +1.25; *p* = 0.049), and defender position (β = +2.40; *p* = 0.021). The adjusted coefficient of determination of the model was R ^2^ = 0.27, indicating that these factors explain approximately 27% of CE variations ( Table 2 ).

**Table 2:** Model for right-sided condylar efficiency as a dependent variable

Variable	Coefficient (β)	*p* -value
Age	-0.38	0.004
Dominance (left side)	+1.25	0.049
Field position: center-back	+2.40	0.021
Adjusted R ^2^	0.27	−

**Fig. 4:**
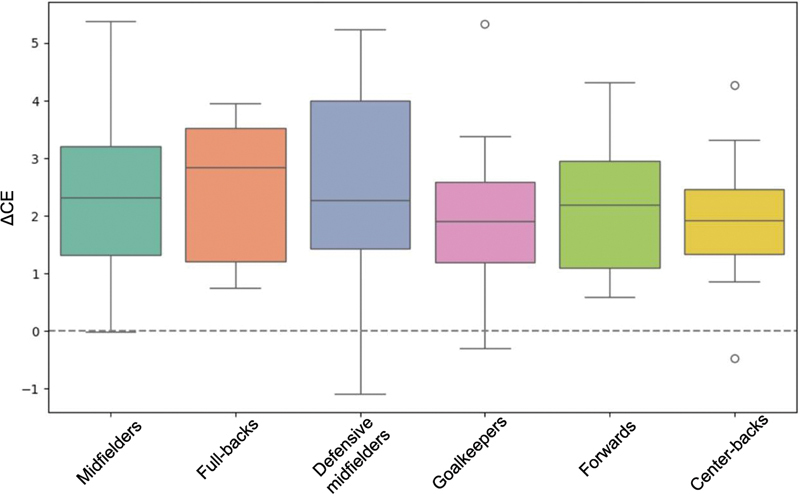
**Distribution of ΔCE according to athletes' playing positions.** Greater variability is observed among full-backs, midfielders, and defensive midfielders.

The MANOVA revealed a significant association between field position and biomechanical variables (Wilks' λ = 0.69; F = 2.42; *p* = 0.0016). Lateral dominance did not show statistical significance (Wilks' λ = 0.92; F = 1.21; *p* = 0.243) ( Table 3 and Fig. 4 ).

**Table 3:** Multivariate analysis of variance

Factor	Wilks' λ	F	*p* -value
Position	0.69	2.42	0.0016
Dominance	0.92	1.21	0.243

The CE was 2.1% higher in the right than the left knee ( *p* = 0.011). The mechanical MPTA (mMPTA, that is, the slope angle of the upper part of the tibia) showed a difference of +1.0° on the right side ( *p* = 0.044). The mechanical LDFA (mLDFA) was 0.7° lower on the right side, with a higher value on the left side ( *p* = 0.033) ( Table 4 ). Regarding differences between sides (Δ), there were significant negative correlations for age and ΔCE (ρ = -0.31; *p* = 0.006) and age and ΔmMPTA (ρ = -0.24; *p* = 0.027) ( Table 5 ).

**Table 4:** Bilateral angular differences calculated as the difference between the right and left sides

Variable	Test	Mean difference	*p* -value
CE (%)	Wilcoxon	+2.1%	0.011
mMPTA (°)	paired t-test	+1.0°	0.044
mLDFA (°)	paired t-test	-0.7°	0.033

Abbreviations: CE, condylar efficiency; mLDFA, mechanical lateral distal femoral angle; mMPTA, medial mechanical proximal tibial angle.

**Table 5:** Correlation of condylar asymmetry (ΔCE) and medial mechanical proximal tibial angle difference (ΔmMPTA) with age

Difference	Spearman's coefficient	*p* -value
ΔCE	-0.31	0.006
ΔmMPTA	-0.24	0.027

**Fig. 5:**
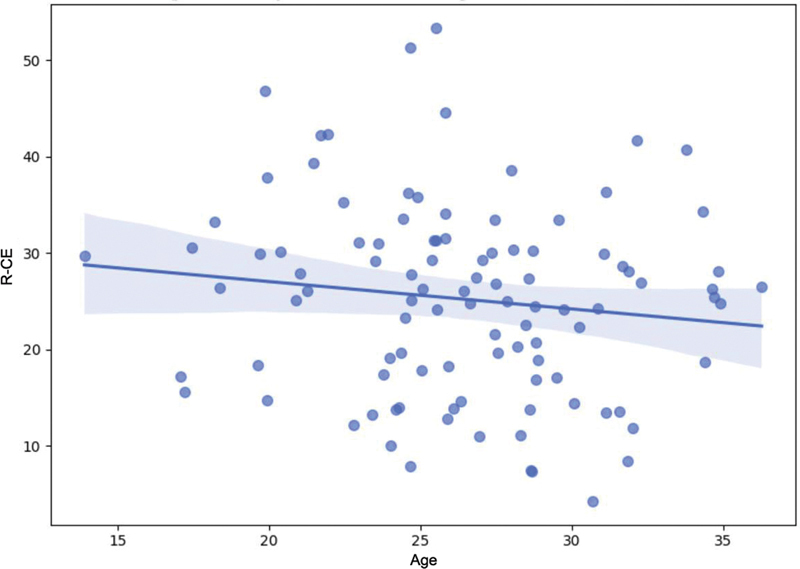
Association between age and right condylar efficiency (R-CE), with a negative correlation and 95%CI.

The multiple regression model with ΔCE as the dependent variable identified the following statistically-significant predictors: age (β = -0.33; *p* = 0.008), indicating less asymmetry in older athletes (β = +1.4; *p* = 0.041), associated with greater asymmetry; and defender position (β = +1.9; *p* = 0.019), which was also related to greater differences. The model presented an adjusted R ^2^ of 0.22 ( Table 6 and Fig. 5 ).

**Table 6:** Predictive model for condylar asymmetry

Variable	Coefficient (β)	*p* -value
Age	-0.33	0.008
Left-sided dominance	+1.4	0.041
Center-back	+1.9	0.019
Adjusted R ^2^	0.22	−

The valgus/varus angle showed a significant difference between sides, with an average of 1.2° on the right and 1.5° on the left side ( *p* = 0.045), suggesting a slight increase on the latter. The mean flexion was of 87.5° for the right side and of 86.8° for the left side, with no significant difference.

Age showed no significant correlation with the right-sided valgus/varus angle (r = 0.15; *p* = 0.12). In contrast, a negative correlation was observed between age and right knee flexion ( *r* = − 0.22; *p* = 0.03), indicating a reduced range of motion in older athletes.

The ANOVA showed a significant difference in the valgus/varus angle between field positions (right side: F [4.95] = 2.89; *p* = 0.027), with full-backs showing higher mean angles. There was no significant difference in knee flexion among positions (right side: F [4.95[ = 1.76; *p* = 0.14).

The independent *t* -test showed no significant difference between right-handed and left-handed subjects for the valgus/varus angle ( *p* = 0.38) and right knee flexion ( *p* = 0.22), indicating that lateral dominance did not influence the joint parameters analyzed.

## Discussion

The current study investigated the relationship involving orthopedic angles of the lower limb and clinical and functional factors in professional athletes, focusing on tibiofemoral mechanical angles and condylar loading, and considering age, field position, and lateral dominance.

### Age and Joint Changes

Age and CE presented a negative correlation, indicating less joint adaptation in older athletes. This finding is consistent with the trend described by Steffen et al., 9 who reported a progressive increase in varus with advancing age and cumulative overload. Davidoviča et al. 10 observed early biomechanical changes in the hip and knee, suggesting that condylar remodeling in professional athletes results from the progression of these adaptations. Aging in sports is associated with reduced CE and changes in the mechanical axis, reinforcing the need for continuous monitoring of these variables.

### Field Position Effects

Field position significantly influenced the evaluated angles (Wilks' λ = 0.69; *p* = 0.0016), with defenders and full-backs showing greater condylar asymmetry. Similar findings were described by Steffen et al., 9 who observed greater varus in defenders, and Ribeiro et al., 11 who highlighted the multifactorial nature of positional adaptations. Murillo-Ortiz et al. 12 also reported asymmetries resulting from repetitive unilateral movements. Thus, tactical function and position-specific load modulate the mechanical axis, promoting osteoarticular adaptations over time.

### Lateral Dominance

The MANOVA revealed no significant overall difference between right- and left-footed subjects. However, lateral dominance influenced the regression models, with left-footed players showing a greater ΔCE, possibly due to the preferential execution of unilateral technical gestures. These findings are consistent with those from Paravlic et al., 13 who reported subtle asymmetries between dominant and non-dominant limbs, attributed to the unilateral repetition of kicks and supports. Murillo-Ortiz et al. 12 also observed small differences in unilateral exercises, reinforcing that dominance and field position generate functional biomechanical adaptations with no pathological character. Thus, the greater ΔCE observed in left-footed subjects reflects functional adaptation to technical dominance, and not structural deformity.

### Lateral Assimetry

There were significant bilateral differences regarding the CE, mMPTA, and mLDFA, more evident in young athletes, suggesting a maturational effect in the pursuit of functional symmetry. These findings are consistent with those made by Steffen et al., 9 who reported a progressive reduction in angular asymmetries with increasing age and sports practice time. The persistence of discrepancies in defenders and left-footed players reinforces the adaptive pattern described by Paravlic et al. 13 and Murillo-Ortiz et al., 12 in which unilateral movements and asymmetrical overload promote specific condylar adjustments. Clinically, such asymmetries, although physiological, may increase the risk of joint overload in contact sports with deceleration and rapid changes of direction.

### Clinical Implications

Our findings reinforce the importance of bilateral monitoring of joint angles, as asymmetries can appear early and evolve with sports practice. Steffen et al. 9 and Paravlic et al. 13 highlighted that systematic monitoring of limb alignment and balance is essential to prevent overload and biomechanical alterations. Murillo-Ortiz et al. 12 reported that preventive interventions should consider field position and lateral dominance to reduce unilateral overload and promote functional symmetry. The CE and mechanical tibiofemoral angle (mTFA) were sensitive indicators of joint overload, enabling the early detection of biomechanical alterations associated with the risk of injury.

### Measurement and Selection Biases

The current study presents potential biases. The inclusion of athletes from a single professional team may limit generalizability, while the exclusion of injured or rehabilitating athletes reduces clinical representativeness. Regarding measurement bias, although the radiographic protocol is standardized, inter- and intraobserver variations in angle measurement may have occurred due to positioning and interpretation, despite the use of digital tools and statistical methods for error reduction. These limitations warrant cautious interpretation of the findings and highlight the need for multicenter, longitudinal studies using automated measurement techniques to improve result validity.

### Limitations and Perspectives

Despite the representativeness of the sample and statistical robustness, the cross-sectional design limits causal inferences and prevents the evaluation of the longitudinal progression of joint angles. Additional variables, such as time spent practicing sports, injury history, and motor preferences, could enrich the models. It is recommended that multicenter and longitudinal studies associate morphological data with clinical outcomes such as pain, injury, and performance.

### External Validity and Result Generalization

The inclusion of athletes from a single professional soccer team in the city of Belém, state of Pará, Brazil, limits external validity by reducing cultural and structural diversity. Although sample homogeneity facilitates control of confounding variables, it restricts generalization to very young athletes, amateurs, athletes from other regions, or female athletes. Therefore, the findings should be interpreted with caution outside this specific context. Nevertheless, the results provide a relevant basis for future hypotheses and underscore the need for broader investigations to enhance the clinical applicability of the evidence.

## Conclusion

Valgus alignment was the most prevalent abnormality (68.62%), predominating among lateral and midanterior athletes, with bilateral valgus occurring in 47.05% players, suggesting greater anatomical symmetry. Varus occurred in (17.64%), mainly unilateral (left: 5.88%; right: 10.78%), indicating asymmetrical functional adaptation. The mechanical axis showed a negative correlation with age, as athletes up to 22 years old had a higher prevalence of valgus (72%), associated with CE > 7° and mLDFA < 88°. Athletes aged 23 years or older presented with a more balanced distribution between valgus (52%) and neutral (43%), suggesting progressive stabilization of alignment with aging. Center-backs and left-footed players presented greater ΔCE, and 68% of the athletes showed an association between the dominant limb and lower CE (< 7°) or mMPTA (> 3°) values. Meanwhile, the supporting limb exhibited greater valgus (CE > 7° and mMPTA > 3°), reflecting specific positional and biomechanical demands. The CE correlated negatively with the mLDFA (-68.8% on the right and -69.4% on the left side) and positively with the mMPTA (+60.6% and +60.3% on the right and left sides respectively), while the mTFA showed an intense negative correlation (-99.7%), evidencing a direct influence on the position of the mechanical axis.
